# Microbial community response to hydration-desiccation cycles in desert soil

**DOI:** 10.1038/srep45735

**Published:** 2017-04-06

**Authors:** Adam Šťovíček, Minsu Kim, Dani Or, Osnat Gillor

**Affiliations:** 1 Ben Gurion University of the Negev Sede Boqer Campus, Zuckerberg Institute for Water Research, Blaustein Institutes for Desert Research, Beer Sheva, 84990, Israel; 2 Soil and Terrestrial Environmental Physics (STEP), Department of Environmental Systems Sciences (USYS), ETH Zürich, 8092 Zürich, Switzerland

## Abstract

Life in desert soil is marked by episodic pulses of water and nutrients followed by long periods of drought. While the desert flora and fauna flourish after rainfall the response of soil microorganisms remains unclear and understudied. We provide the first systematic study of the role of soil aqueous habitat dynamics in shaping microbial community composition and diversity. Detailed monitoring of natural microbial communities after a rainfall event revealed a remarkable decrease in diversity and a significant transition in community composition that were gradually restored to pre-rainfall values during soil desiccation. Modelling results suggest a critical role for the fragmented aqueous habitat in maintaining microbial diversity under dry soil conditions and diversity loss with wetting events that increase connectivity among habitats. This interdisciplinary study provides new insights into wetting and drying processes that promote and restore the unparalleled microbial diversity found in soil.

Natural and highly dynamic variations in soil hydration conditions shape life in arid soil. Especially, soil microbial life is critically dependent on the amount and availability of water in pores and liquid films on surfaces^1^,^2^ as aqueous phase configuration determines nutrient diffusion and microbial dispersion ranges^3^,^4^,^5^. Furthermore, the temporal dynamics of water configuration within soil matrix due to rainfall, drainage, and evapotranspiration affect microbial community composition and function^6^,^7^,^8^, most critically in water limited desert soil^9^,^10^.

More than 35% of the Earth’s terrestrial surfaces are permanently or seasonally arid^11^ where water is replenished by infrequent rainfall. Soil water is the main limiting resource for soil primary and secondary production and thus variable precipitation pulses in arid ecosystems boost massive vegetative growth^12^,^13^. These bursts of primary productivity and the subsequent increase of plant biomass and diversity in deserts are well documented^14^; but how do soil communities of microorganisms respond to the sudden availability of water?

The effects of episodic wetting of arid soil on the diversity, size and function of microbial communities remain unclear. Early studies suggested that rainfall entails an increase in biomass and enhanced microbial activity^6^,^7^,^15^ similar to that which occurs with macro-organisms. The carbon source for the microbial propagation was postulated to be either a portion of microbial biomass that died under dry conditions^16^ or large amount of microbial biomass that perishes during early stages of wetting due to osmotic shock^6^,^16^, in contrast, it was also suggested that the carbon originates from osmolytes released by cells during desiccation^17^. Regardless of its origin, the microbial remnants would provide nutrients to the rain-activated communities, hence triggering CO_2_ pulse and boosting new biomass^16^.

More recent studies that applied the tremendous advent of DNA analyses, have challenged these assumptions suggesting that the soil microbial response to wetting is independent of biomass^9^,^17^,^18^. In conjunction with a stable biomass, a massive release of CO_2_ was documented after abrupt wetting of desert soils and has been suggested to result from an increase in microbial metabolic activity^8^,^17^. This increase in activity could enhance microbial diversity, due to the positive correlation between microbial productivity and the length of the microbial food chains^19^ or due to increased bacteriophage activity that keeps copiotrophs at bay and support diverse microbial communities^20^.

The response of microbial communities to hydration and desiccation could reflect changes induced by their varying environment. Concurrent changes in habitat connectivity^4^,^21^, and temporal dynamics of nutrients diffusion or microbial dispersion ranges and pathways^22^. Increase in microbial dispersal rates and ranges could either increase richness by migration that could sustain and enhance the population, or decrease richness by diminishing local refuges that served competitively weaker taxa^23^.

Most studies on soil wetting effects on microbial diversity, to date, have concentrated on the hydration events following long droughts, often ignoring the role of subsequent desiccation^10^,^24^,^25^. The few studies that examined the effects of soil water on residing microbial communities and the gradual drying that follows a rain event suggested that initial microbial diversity would eventually re-establish^24^,^26^,^27^. However, the patterns and dynamics of arid soil microbial communities during a hydration-desiccation cycle have not been previously studied systematically. Furthermore, the impact of rapid wetting on microbial community characteristics through a shift from oxic to anoxic condition has rarely been quantified. We predicted that water pulse following a long drought would modify the soil microbial habitats invoking changes in the microbial community composition. However, we inferred that the overall diversity would remain constant as adapted members of the microbial community would quickly occupy newly emerging niches.

The study describes the temporal dynamics of desert soil microbial diversity and community composition monitored in the field before, during, and after a rain event. Furthermore, a mechanistic model was developed to simulate changes in aqueous habitats and microbial community composition following the wetting and drying event including the onset of anoxic conditions.

## Results

### Community composition changes during a rainfall event: Field observations

The cumulative rainfall during the field study reached ~35 mm in the course of two days^28^. The water content of the soil collected before the rain event (September 2012) was about 1% (g/g) which is equivalent to matric potential of −10 MPa (calculated according to Castelblanco *et al*.^29^). During two rainy days, the soil water content increased to 17% (g/g) (Figure S4) with total rainfall depths of ~16 mm and ~19 mm for the first and second consecutive rainy days, respectively. This water content is equivalent to a matric potential of −10 kPa and a degree of saturation ~0.6 suggesting the soil reached its water holding capacity. Observations have shown a gradual desiccation of the soil over time after the rain. Detailed chemical analysis of the soil solution did not reveal significant changes except in the total ammonia and total nitrate (see Figure S1). The amount of total nitrate increased right after the rain and gradually decreased as soil desiccated while the amount of total ammonia exhibited complementary behaviour with nitrate.

**Figure 1:**
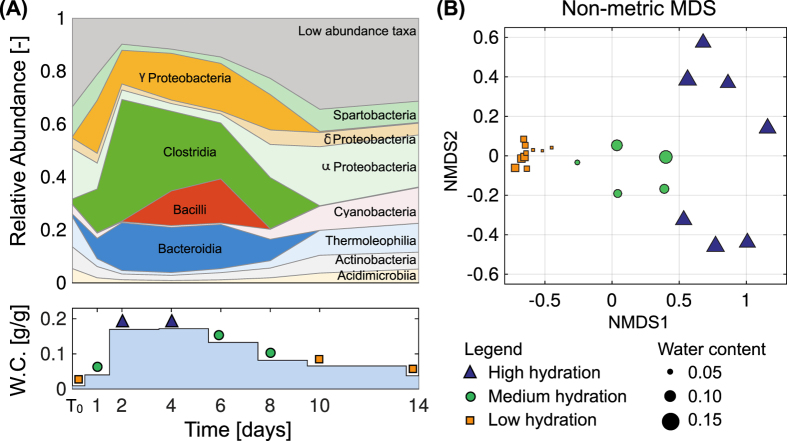
(**A**) The dynamics of relative abundance of soil microbial classes during the field observations. Each time point is an average of three biological replicates. Time zero is an average of three samples taken from fully desiccated soil during the summer of 2012. (**B**) Non-metric Multidimensional Scaling based on the soil rRNA sequencing (MiSeq) dataset. The size of the symbol corresponds to the water content measured in the soil at the time of sampling.

We applied qPCR analysis on extracted ribosomal RNA showing that the total abundance of microbial ribosomes was stable over the wetting-drying cycle. We quantified the abundance of microbial ribosomes throughout the wetting and desiccation period in all our soil samples (see Figure S2). No significant changes in the total ribosomal count was detected except in the first sample taken during severe desiccation, which showed an order of magnitude decrease in ribosomal count. While the total abundance remained stable, the relative abundance exhibited dynamic changes following the rainfall event (Fig. 1A). Observed is that several taxa became dominant for the first few days after the rainfall (*Enterobacterales, Clostridiales, Lactobacillales* and *Bacteroidales*). Subsequently, these dominant classes slowly declined in the ensuing soil desiccation. Some of these, specifically *Clostridiales, Lactobacillales* and *Bacteroidales* are known to include many anaerobic species^30^ implying that some niches in the saturated desert soil have become anaerobic. Importantly, the pre-rainfall community composition was recovered within six to seven days as the soil water content dropped below 10% (g/g).

The changes in the relative abundances of soil microbial taxa reflect drastic and rapid changes in microbial community composition. To track the shift in the microbial communities during the wetting and drying cycle, we performed a non-metric multidimensional scaling (Fig. 1B). Three distinct communities emerged: one (orange squares) consisting of a microbial community in desiccated desert soil (low hydration conditions), a second (blue triangles) clustering the communities in very wet soil (the saturation degree is about 0.5~0.6, denoted as high hydration conditions), and a third (green circles) banding together a community during the gradual desiccation (denoted as medium hydration conditions). Moreover, soil communities were grouped according to time of sampling: samples collected during low hydration conditions (time zero, days 1, 8, 10 and 14), high hydration (days 2, 4 and 6) and intermediate hydration conditions (days 6 and 8), formed separate clusters, quantitatively. This implies that the main component of the community before the rain event and after the desiccation was not affected.

### Community diversity metrics during a rainfall event: Field observations

**Figure 2:**
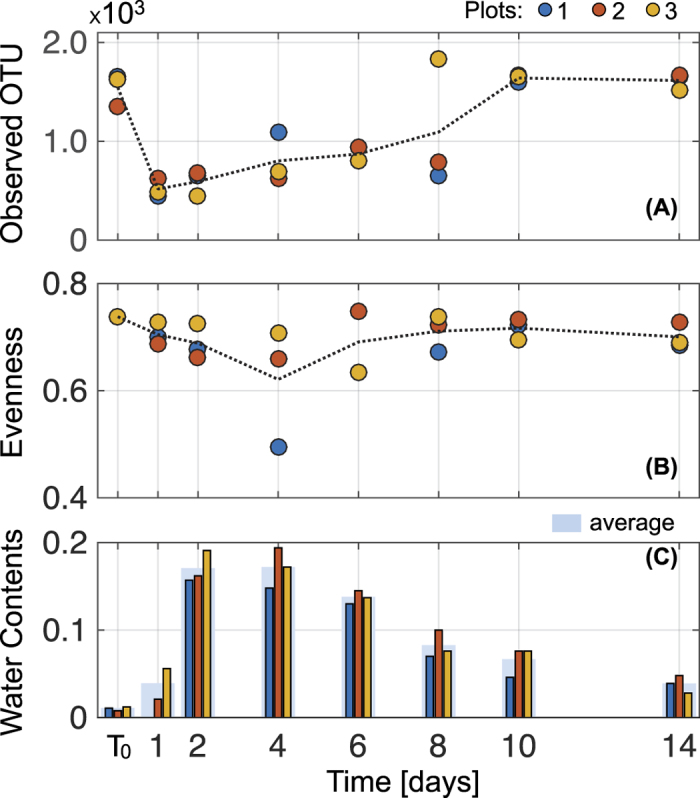
Richness and evenness of soil bacterial populations sampled in three adjacent plots during a wetting-drying cycle. Population richness was expressed as the number of observed species (**A**) and population evenness is displayed using Pielou’s evenness index (or Shannon’s evenness) (**B**). The trend line is the averaged value of the data. The measured gravimetric water content of each plot (in corresponding colours) and the averaged values are given in (**C**).

The variations in relative abundance were translated to changes in soil microbial diversity. Figure 2 depicts the dynamics of field measured microbial diversity during the wetting-drying event in terms of richness, observed OTU, and evenness, Pielous’s evenness. In Fig. 2A, the richness index indicated a statistically significant drop during the rain event (days 1 and 2) and initial stages of desiccation (days 4 and 6) (per group *t* < −11.80, per group *p* < 1.8 × 10^−10^, Figure S5, Figure S6, SI Text 2). Similarly, we observed a slight decrease in evenness, but unlike changes in richness, the evenness index displayed gradual but statistically significant changes (field observation *F* = 77.8, *p* = 3.015 × 10^−6^, Figure S5, Figure S6, SI Text 2) and steady recovery during the hydration cycle (Fig. 2B). In the field, we monitored three adjacent plots concomitantly; minute differences in their desiccation rates instigated observable differences in the community (Fig. 2C). For example, the soil in plot 3 (marked as yellow in the figure) dried relatively faster than in plots 1 and 2, possibly leading to earlier onset of changes in microbial diversity in the plot.

### Microbial community dynamics during a rainfall event: model predictions

**Figure 3:**
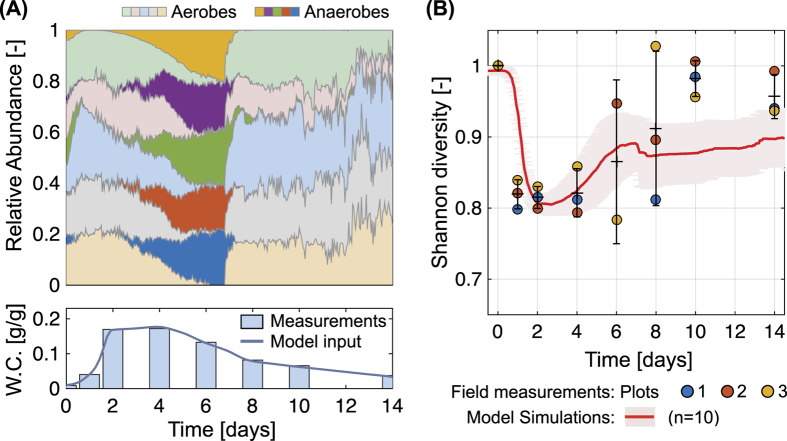
A wetting-drying cycle was applied to the modified rough-surface patch model (RSPM) to observe its effect on bacterial diversity and the community composition. (**A**) The relative abundance dynamics of modelled bacterial classes are depicted. In the simulations, 40 virtual taxa (20 taxa growing aerobically and other 20 taxa growing anaerobically) were inoculated. For this figure, the virtual taxa were classified to 10 classes (5 aerobic groups shown in light colours and 5 anaerobic groups shown in dark colours) and the relative abundance dynamics of 10 independent simulations were averaged. (**B**) Shannon index of the simulated bacterial populations were compared with the field measurements suggesting that in both the diversity decreased rapidly after wetting and then recovered with drying. For this comparison, the field measurements and the simulation results were rescaled with the value at day 0 (dry soil) indicating the relative changes. Results from 10 individual simulations (with different inoculation of microbial cells and different soil structures with same pore size distribution and porosity) are averaged (red line) and shaded area (in pink) indicates the standard deviation (s.t.d.). Averaged values of measurements are presented with mean ± s.t.d. (black solid lines) calculated from three environmental replicates in 3 adjacent plots.

We applied a mechanistic model for microbial populations’ dynamics during changes in soil wetness (induced by the rainfall event). The model results were in qualitative agreement with field observations in terms of microbial diversity and the community composition changes. Figure 3 depicts the predicted effect of hydration dynamics on the soil microbial community. After soil wetting, the relative abundance of various taxa exhibited a dynamic response. Figure 3A shows the rise of anaerobic classes (marked with strong colours) from day 2 to day 7, replacing aerobic classes that were prevalent in the dry soil. The model results show the sharp decrease of anaerobes at between day 6 and day 7 as the soil became aerated again. While drying, air penetrates through the profile and shifts most of the domain back from anoxic to oxic conditions (corresponding to a water content of 0.1 [g/g]). Furthermore, in Fig. 3B, the relative changes in Shannon index was chosen as the key diversity index for a comparison with the field observations. The decrease in diversity indicates the rises of dominant taxa during wetting. This is driven by competitive interactions among individuals over a common substrate, in this case the carbon source. The connected aqueous habitats and the increased dispersal of cells allowed intense competition for the substrate thereby causing the changes in diversity. The recovery of diversity reflects the role of aqueous habitat fragmentation. As the degree of connectivity in the aqueous phase decreased, microbial interactions are spatially limited^31^. Essentially, the observed dynamics of community composition and diversity are the outcome of simultaneous effects of the competition over dissolved substrates and the temporary dominance of anaerobic taxa due the transition from oxic to anoxic conditions in some parts of the wet soil.

## Discussion

Several studies have followed soil microbial communities *in situ* measuring their diversity and community composition following wetting events^21^,^32^,^33^. The findings generally support higher microbial diversity (or coexistence degree) under drier conditions where the aqueous-phase is largely fragmented and dispersion is limited. In previous studies, hydration conditions were under controlled laboratory experiments^9^,^10^,^17^,^24^, and in the field following the first rainfall after a prolonged draught^18^,^24^,^25^. In contrast with such a step-change in hydration conditions, the dynamics of soil microbial diversity during a cycle of wetting and gradual desiccation received little attention. Here, we provide a detailed account of microbial response to wetting and subsequent drying in desert soil in the field and using a mechanistic model.

We quantified soil microbial community composition in desert before, during, and after a major rain event. Microbial community dynamics deduced from field observations were compared with results of the mechanistic model that simulated substrate diffusion and growth of multi-taxa microbial communities on idealised hydrated soil profile. The field observations and modelling results yield similar temporal dynamics of microbial diversity and community composition during hydration-desiccation cycles. The results show a significant change in composition together with a decrease in soil microbial diversity upon wetting, and gradual recovery as the soil dries to pre-rainfall levels (Figs 1,2 and 3). The model focuses on the putative role of aqueous phase connectivity. Following soil wetting, the increased connectivity of habitats facilitates higher rates of substrate diffusion and larger ranges of cell dispersion as key mechanisms for the observed loss of diversity during wetting^31^,^34^. Furthermore, the detailed account of water configuration dynamics in the soil profile and associated oxygen diffusion suggest the possibility of establishing anoxic conditions following wetting that may last a day or two within the soil volume.

We have shown that microbial community composition and diversity co-vary during wetting, and subsequently recover to pre-wetting levels as the soil desiccates (Figs 1 and 2). Notably, the extensive changes in diversity were not reflected in the abundance of active microorganisms (Figure S2), this is in agreement with previous studies that show no changes in soil bacterial abundance with hydration following a long drought^9^,^10^,^18^. The soil community composition was significantly altered after a large rainfall event (Fig. 1). Some of the observed changes under field conditions could have resulted from dispersal and establishment of other migrated taxa during desiccation. Yet, in the model such dispersal processes were minor, and the resulting diversity patterns were similar to field observations (Fig. 3). Moreover, the nMDS suggest that the community composition had returned to pre-rainfall composition (Fig. 1B). This supports that immigration effect during and after wetting is questionable. We thus conclude that although dispersal and migration could contribute to changes in community composition, they are probably not the main factors in this complex ecosystem.

A factor that may have contributed to the changes in microbial community composition was the formation of anoxic regions in the soil following the rainfall^35^,^36^. In other words, the high hydration conditions near the soil surface and the stimulation of biological activity limited oxygen diffusion and promoted favourable conditions for anaerobic organisms (Figs 1 and 3A). Such episodic increase in anaerobic taxa with the onset of anoxic conditions has been shown in previous studies^37^ and their occurrence was coincidental to increase fluxes of carbon and nitrogen^35^,^38^,^39^. The carbon flux is introduced with precipitation from the carbon fixing soil crust in this system^40^. This results in an increase of available organic carbon in the top soil profile during days 1–3 (Figure S1g). This was represented in the model by the substrate entering to the system given as the constant concentration boundary condition on the top of the domain. Mass flux, however, is elevated during wetting due to greater water film thickness (See SI Text 4). Heterotrophic organisms utilise this fixed carbon source for their activity. Since metabolism in anoxic environments requires different terminal electron acceptors, nitrate respiration is expected. The soil nitrate pool increases during dry periods and this will be available for anaerobes together with carbon sources right after the wetting^40^. Changes in total nitrate (Figure S1d) support the postulated increase in anaerobic activity^40^. On the other hand, total ammonia shows a complementary behaviour to nitrate (Figure S1b) owing to the suppressed aerobic activity as aerobic metabolism requires ammonia as the nitrogen source. We note that it is not clear where the ammonia flux comes from. It could be either nitrogen fixing bacteria on the crust or a deposited source at the surface introduced by the rainfall event. Although nitrogen dynamics were not considered explicitly in the model, the observed community dynamics match with the predictions. This implies that the sampled area might be mostly carbon limited during the wetting-drying cycle as the model assumed.

The generally dry and aerated arid soil conditions suggest that such dominance is limited to narrow windows of hydration conditions after major rain events and before the soil dries. Within this narrow activity window in the field, the community richness has markedly decreased (Fig. 2A) and the diversity patterns were consistent in both the field and the model (Figs 2 and 3B). The rise of putative anaerobic taxa replacing aerobic taxa in the dry soil does not necessarily explain the decrease in diversity as it could have temporally increased richness during the transition from oxic to anoxic or vice versa. This suggests that oxygen depletion might not be the main driver for the observed decrease in diversity.

A full representation of microbial diversity found in soil is beyond the computational capacity of most models. Predicting microbial responses from dynamic hydration conditions in soil might be even more challenging. The model attempts to minimise the variability of physiological differences among taxa and to highlight the effect of physical and chemical processes in soil during wetting and drying. Dividing microbial taxa in soil community to only two groups, obligate aerobes and obligate anaerobes, is somewhat arbitrary, yet imposing the field-measured hydration values yielded a reasonable time scale for anoxic dominance. The sharp transition in community composition in the model predictions (Fig. 3A) reflects on immediate inhibition of anaerobic activity following the gas phase percolation (increase in oxygen concentration). In reality one expects a more gradual response afforded by soil heterogeneity and a range of microbial responses to the presence of oxygen. For example, including facultative anaerobes or aerotolerant anaerobes might form groups which persist through out the wetting-drying cycle and smoothen the transition.

The model distinguishes microbes according to the response to O_2_ presence, as well as a substrate utilisation through Monod parameters (specific growth rate and affinity to the carbon source), while other morphological and physiological characteristics were assumed to be identical. Each combination of parameters defined a “taxon” and was counted in the diversity metrics. The choice for the parameter range can change the intensity of diversity response in the model, yet the impact is relatively small and the selected values cover the realistic range^41^. While the model predicts the response in Shannon index by tracking the microbial populations, the observed decrease in richness was not met. This is because of the dominance of rare members in the real community indicating that other survival strategies may exist besides the growth functions (i.e. facultative anaerobes, aerotolerant anaerobes, or microaerophiles). Furthermore, the diurnal changes in the soil temperature were also included in the model of microbial activity, but its contribution were not significant, since the variation of the soil temperature keep within the range of 2–5 °C (See SI Text 4).

Despite numerous simplifying assumptions of taxon numbers and their interactions, the model qualitatively reproduces the community dynamics observed in the field (Figs 2 and 3). In contrast with our initial hypothesis we noted drastic changes in both the diversity and the composition of soil microbial communities after soil wetting. The interplay between the sudden increase in nutrient fluxes, rapid dispersion and stronger competition over the dissolved carbon source was important for the diversity dynamics. The enhanced connectivity during wetting resulted in the reduction of diversity with small effects on nutrient-consumption patterns while altering the composition from the aerobes-dominated to anaerobes-dominated community. The remarkable recovery of diversity and composition with the onset of dry conditions illustrates the resilience of the microbial communities in desert soils following major rain events. The field results together with the model simulations point to the centrality of the configuration of water and gas in soil shaping cell dispersion and nutrient diffusion and thus in microbial community diversity and composition in a highly dynamic soil environment.

## Methods

### Soil-sampling scheme

The field survey was performed in the morning, by sampling barren patches at the long-term ecological research (LTER) station of Avdat (30° 47’ N, 34° 46’ E, 600–700 m elevation), situated in the central Negev desert, Israel. The sampling sites were selected as replicate fields with comparable soil physico-chemical parameters **(unpublished data)** and vegetation coverage^42^.The average mean annual precipitation is ≈90 mm, distributed through several unpredictable rain events during the winter months. The annual potential evaporation is ≈2600 mm^43^. The soil in the sampling site is wind deposited loess^44^ with maximum water holding capacity of 16% (w/w)^45^. Soil samples were collected before, during and after the first major rain event (≈35 mm of rain) in the winter of 2012/13 (Figure S4). We note that the rain event was isolated and was not proceeded or exceeded by minor rain events within four weeks of the recorded event.

Soil chemical analyses followed standard methods^46^ as elaborated in the Supplementary data (See Figure S1). Changes in soil chemical composition and their effect on bacterial diversity were evaluated using linear models (See Figure S6).

Approximately 1 kg soil was sampled between 9 and 10 am from the top 5 cm of the soil profile after discarding the soil crust, using an ethanol-cleaned scoop. At each time point, three composite samples, consisting of eight randomly selected subsamples, were collected from adjacent 40 × 25 m plots (max. distance 20 m), providing three biological replicates. The soil samples were collected into sterile bags, transported to the laboratory and kept at 4 °C until processing (within no more than 4 h of sampling). Soil was homogenized by sieving through autoclave-sterilized sieve (2 mm pore grid size) as previously described^47^. Samples for RNA extraction were collected and stored at −80 °C and the rest of the sample was dried at 65 °C in preparation for the chemical analysis.

### RNA extraction and reverse transcription

The RNA profile of bacterial community was followed due to previously reported DNA recalcitrance in soil environment^48^, especially in response to changes in water content^49^. Total nucleic acids were extracted with phenol/chlorophorm at the pH 7 according to the protocol published by Angel (2012)^47^. Complementary DNA was generated by reverse transcription, using ImProm-II reverse Transcription System (Promega, Madison, WI). The cDNA used for Illumina sequencing was generated using 50–100 ng of the template RNA to ensure a sufficient amount of DNA material for sequencing. In contrast, sequences used for qPCR were generated from 1 ng RNA to ensure precision of the back calculation.

### Quantitative PCR (qPCR)

Total nucleic acids were extracted from the soil samples as previously described^47^. The extract was purified by MasterPure RNA Purification Kit (Epicentre, Madison, WI). The DNA was degraded by DNase I supplied with the kit and the RNA samples were stored at −80 °C for further analysis. All qPCR reactions were performed in an iCycler thermocycler equipped with a MyiQ detection system (Bio-Rad, Munich, Germany). Data were processed using CFX Manager 3.0 software (Bio-Rad). Standards containing a known number of copies of the target gene, 16S rRNA from Escherichia coli, were serially diluted to calibrate each qPCR. The universal primers S-D-Bact-0341-a-S-171 and S- Bact-0515-a-S-19 (Table S1) were used for amplification. Each qPCR contained the following mixture: 10 *μ*l SYBR Absolute Blue qPCR Rox Mix (Thermo Scientific, Waltham, MA), 1 *μ*l of 400 nM of each primer (Metabion, Rehovot, Israel), 5 *μ*l template cDNA and 3 *μ*l molecular-grade water (HyLab, Rehovot, Israel). Abundance estimation was performed under the following conditions: 95 °C for 15 min, followed by 35 cycles of 95 °C for 10 s, 60 °C for 15 s and extension at 72 °C for 30 s.

### Taxonomic analysis

The microbial 16S rRNA units were sequenced with MiSeq (Illumina, San Diego, CA), using primers S-*-Univ-0515-a-S-19 and S-D-Bact-0787-b-A-20 targeting the V3 and V4 region of the gene (Table S1). The resulting reads were clustered into operational taxonomical units (OTUs) that corresponded to 90% of the rRNA difference using the Open reference picking pipeline, combining clustering against a database (Silva 111) with de novo clustering of yet unknown taxa, provided by the QIIME package^50^. OTUs were identified and analyzed, using the Silva 111 dataset^51^ and sequences that were not present in the database were clustered de novo (see SI Text 3). OTU counts in different samples were adjusted equal depth of sequencing (in our case 7955 sequences).

### Non-metric multidimensional scaling

To evaluate the grouping of different communities, we performed a nonmetric multidimensional scaling (nMDS) transformation using the vegan package^52^,^53^. The solution was reached after 5 iterations with the final stress equal to 0.055 (indicative that the transformation is a good representation of the dataset).

### Modelling microbial growth on hydrated surfaces

To assess the trends and soil microbial community dynamics in response to hydration desiccation cycles, we employed a mechanistic model comprised of three major components: representation of the physical soil environment and water and gas configurations, quantification of microbial community functioning at the cell level, and the resulting biophysical interactions during changes in hydration. We have modified previously developed rough surface patch model (RSPM) to represent the physical environment in soil profile that mimics effects of hydration on water films and spatio-temporal changes in nutrient diffusion fields and oxygen inputs from gas phase to liquid phase that support growth of microorganisms^34^. Microbial activities of community members were represented by the Individual-Based Model (IBM) that considers a simple metabolism, motility, chemotactic behaviour and trophic interactions among individuals (See SI Text 4).

The physical domain mimicking soil profile was generated and was inoculated with populations of 40 different virtual microbial taxa. In the model, we considered only 40 taxa to represent population dynamics and their dispersal within the domain (far less than the richness in real soil). The dissolved oxygen concentration was assigned from the partial pressure of the oxygen in the atmosphere (20.09%). Its input to the liquid phase is calculated following Henry’s law and diffuses through the domain from the top of the domain. The concentration of other primary substrate, we assume in this study as the carbon source (e.g. glucose), was fixed at the top boundary of the domain to represent diffusive influx of nutrients into the system. The measured water contents in the field were mapped to the matric potential as a boundary condition of hydration to determine the physical configuration of water and gas in soil matrix. (For the details of its physical, chemical, and environmental conditions that are used as boundary conditions, see SI Text 4). This time-dependent-hydration condition imposed on the domain modifies the nutrient-diffusion field and gas-liquid configuration and thus changes the carrying-capacity distribution over time. The obtained platform simulated the dynamics of the 40 microbial populations during the wetting-drying cycle and this enabled us to calculate the relative abundance dynamics and estimate the Shannon index.

To simplify the representation of a diverse microbial community, firstly, we designed microbial taxa with two distinctive groups, aerobically growing cells and anaerobically growing cells (20 taxa for each group in this work). Here, we assume that the majority of community members are heterotrophic organisms utilising the available carbon source for their metabolism. The growth functions for these two groups are given by different Monod parameters according to:


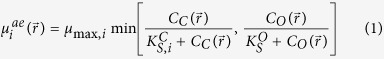



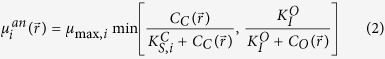


where 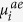 and 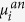 are growth functions of aerobes and anaerobes, respectively. Two groups differentiate with the growth response to the local oxygen concentration, 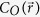, assigned with the half-saturation constant, 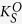, for aerobes and the inhibition constant, 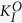, for anaerobes. We note that we did not consider the variation of bacterial response to oxygen and only obligate aerobes and obligate anaerobes are included in the model for the simplicity^54^. 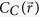 is the local concentration of the carbon source and differences between virtual taxa are assigned with the maximum growth rate and Monod half-saturation on carbon source, 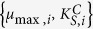. These values were drawn from a wide range of reported literature values of aerobically and anaerobically growing cells^41^,^55^,^56^. However, we note that higher maximum growth rates of anaerobes were assumed to compensate the strong inhibition term of oxygen in the model. The range of Monod parameters that used in the current modelling work are reported in Table SI. 2 in SI Text 4 in Supplementary information. Differences in assigned Monod parameters implied different nutrient-consumption patterns and ecological strategies, spanning the range from “pseudo-copiotrophic” to “pseudo-oligotrophic”, during the hydration cycle^57^. Other properties, such as cell size, shape, motility, chemotactic sensitivity, and optimal temperature for growth were assumed to be equal for all taxa. The substrate yields and maintenance rates were assumed to be different between aerobic taxa group and anaerobic taxa group considering that high costs of anaerobic activity^55^,^56^.

The simplicity gained by pre-assigned Monod parameters enabled definitive tracking of the dynamics of relative abundance and microbial diversity. To avoid loss of taxa during the hydration desiccation cycles (due to the smallness of the community size that could be practically modelled), we tagged the cells at starvation condition (negative growth rate, costs for maintenance exceed growth related costs) as potentially active cells so it contributes to the diversity index dynamics.

### Measuring microbial diversity

We selected the observed OTUs, *N*_*s*_(*t*), as the species-richness estimator *R(t*), and the Pielou’s evenness index as an estimator of population evenness *E*_*P*_(*t*)^58^.


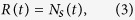



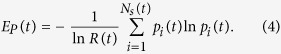


where *p*_*i*_(*t*) is the relative abundance or the probability that a certain individual belongs to species *i* at time *t*. The Shannon Index is the non-normalised form of Pielou’s index estimating the total-diversity;


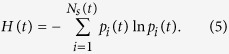


To compare field observations with and model predictions we used relative changes of the Shannon index, 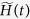 by dividing the data sets by the Shannon index at the onset of the rain event, *t* = 0;


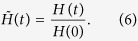


## Additional Information

**How to cite this article:** Šťovíček, A. *et al*. Microbial community response to hydration-desiccation cycles in desert soil. *Sci. Rep.* **7**, 45735; doi: 10.1038/srep45735 (2017).

**Publisher's note:** Springer Nature remains neutral with regard to jurisdictional claims in published maps and institutional affiliations.

## Supplementary Material

Supplementary Information
