# Supplementary material for: Microbial community response to hydration-desiccation cycles in desert soil
Source: Sci Rep. 2017 Apr 6;7:45735. doi: 10.1038/srep45735 (PMC5382909; doi:10.1038/srep45735)
Supplement: Supplementary Information [file srep45735-s1.pdf]

Microbial community response to hydration-desiccation cycles in desert soil Supplementary information

Adam Šťovíček, Minsu Kim, Dani Or, and Osnat Gillor

Figure S1: Chemical analysis

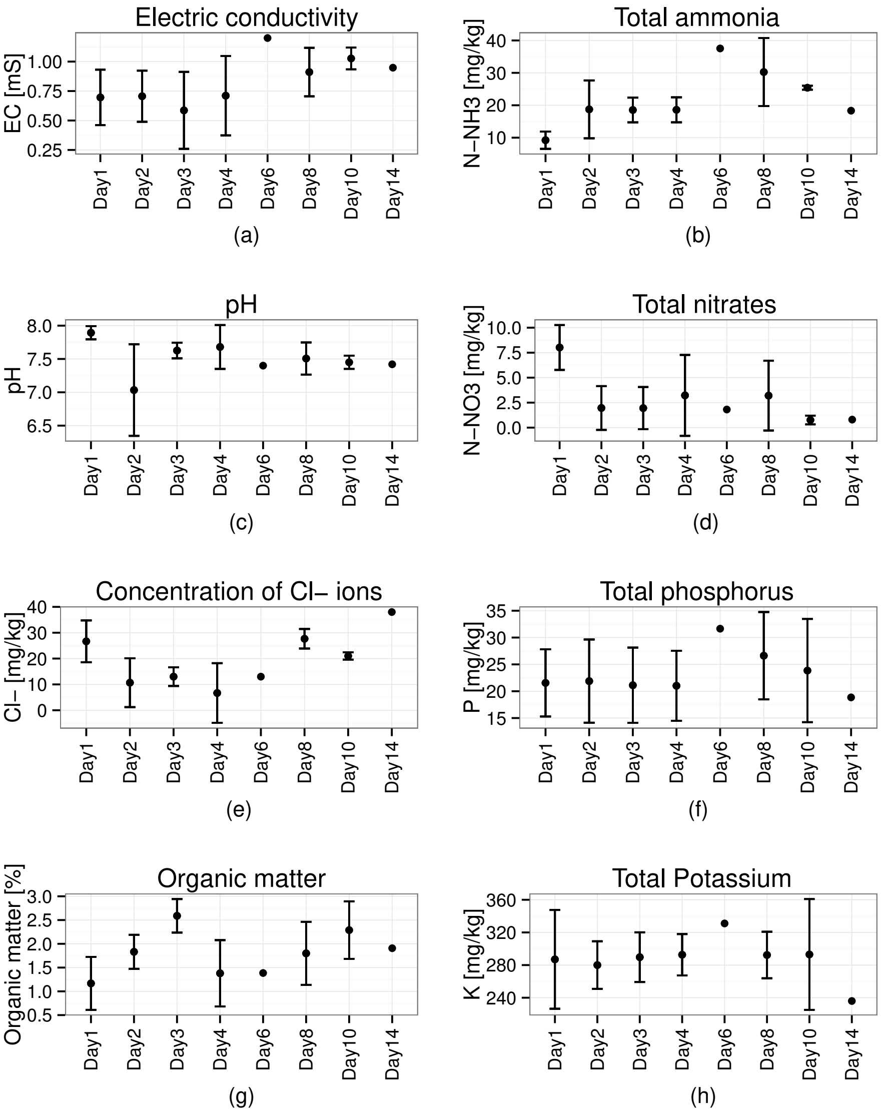

Figure S1. Chemical analysis of the soil samples from the field.

Figure S2: Total bacterial ribosomal count

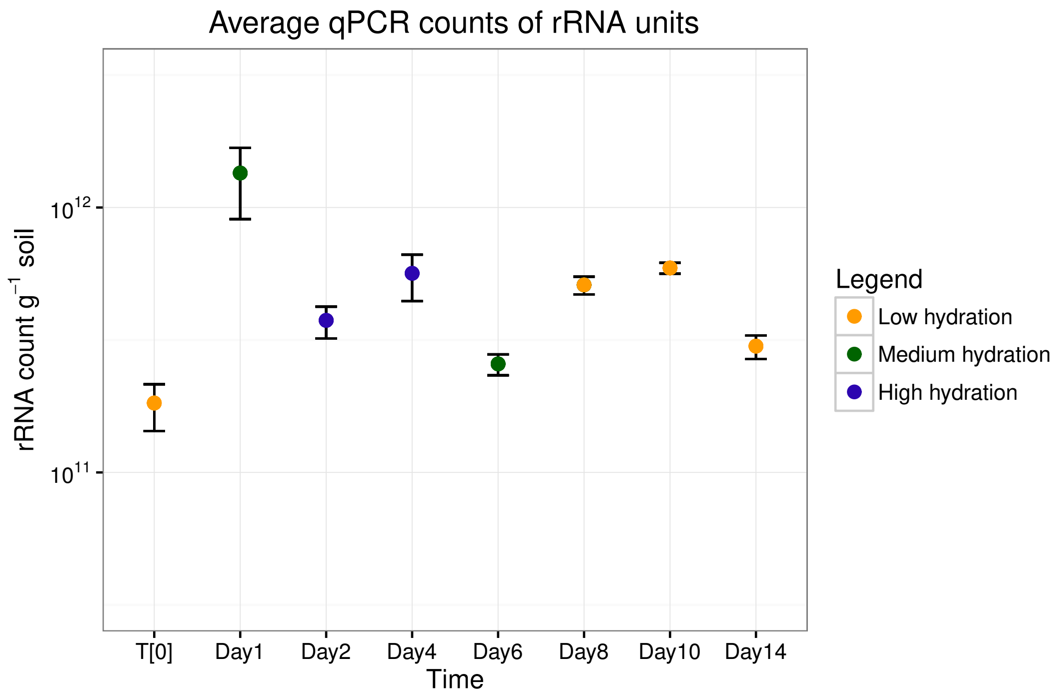

Figure S2. Total nucleic acids were extracted from the soil samples as previously described (Angel 2012). The extract was purified by MasterPure RNA Purification Kit (Epicentre, Madison, WI). The DNA was degraded by DNase I supplied with the kit and the RNA samples were stored at -80°C for further analysis. Total bacterial rRNA unit quantification in the field measurements. Colour coding shows the different categories of samples with respect to community clustering (see Figure S1B in the main text).

Figure S3. Analysis of similarity (ANOSIM) test.

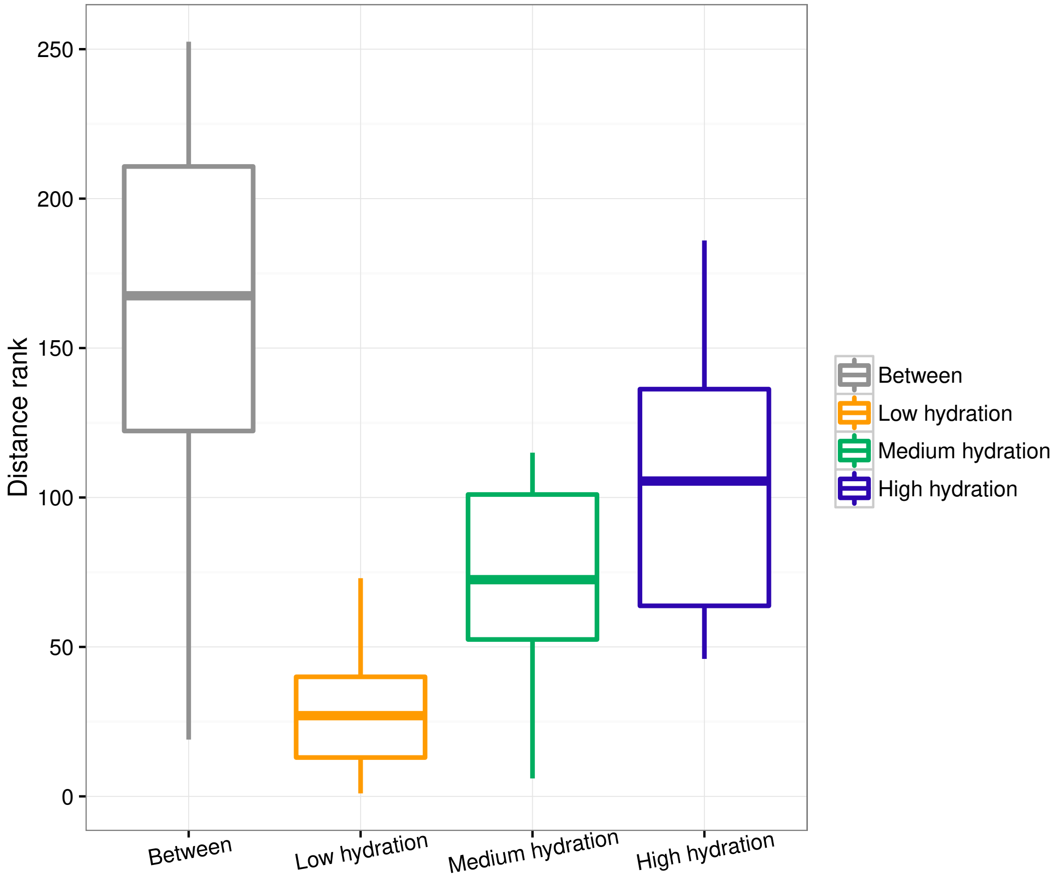

Figure S3. Result indicating statistically significant difference between the four observed NMDS clusters in Figure S3. Consequently, the resulting significant values of R and p support rejection of the null hypothesis that these were a result of a random process.

3 **Figure S4: Soil water content**

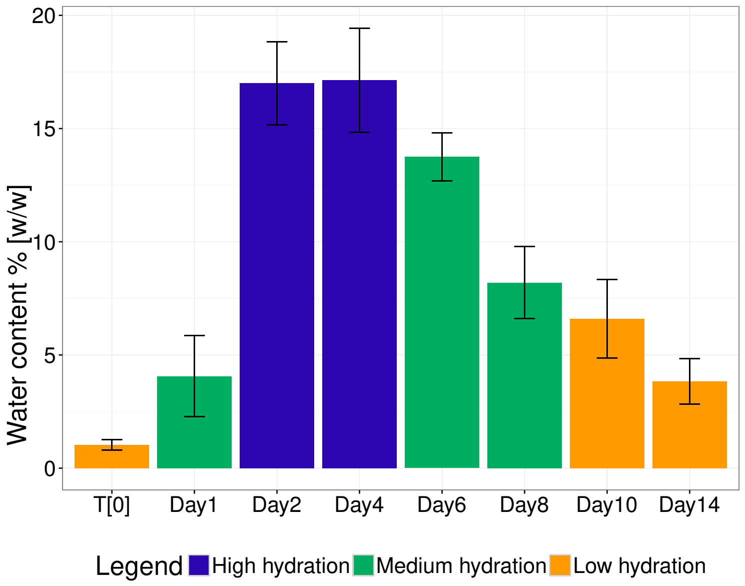

Figure S4. Average water content as obtained gravimetrically during field measurements (N=3).

Figure S5: Richness and evennes per category

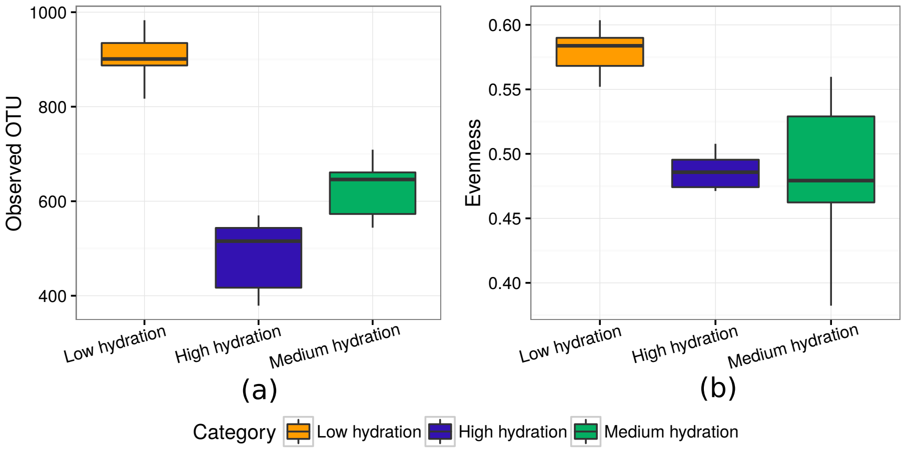

Figure S5. Comparison of the richness (a) and evenness (b) by the wetting categories. Boxes represent upper and lower quartile and the ticks display 5 and 95 quantile$\hat{\mathrm{E}}$($n \geq 3$).

Figure S6: Linear model results

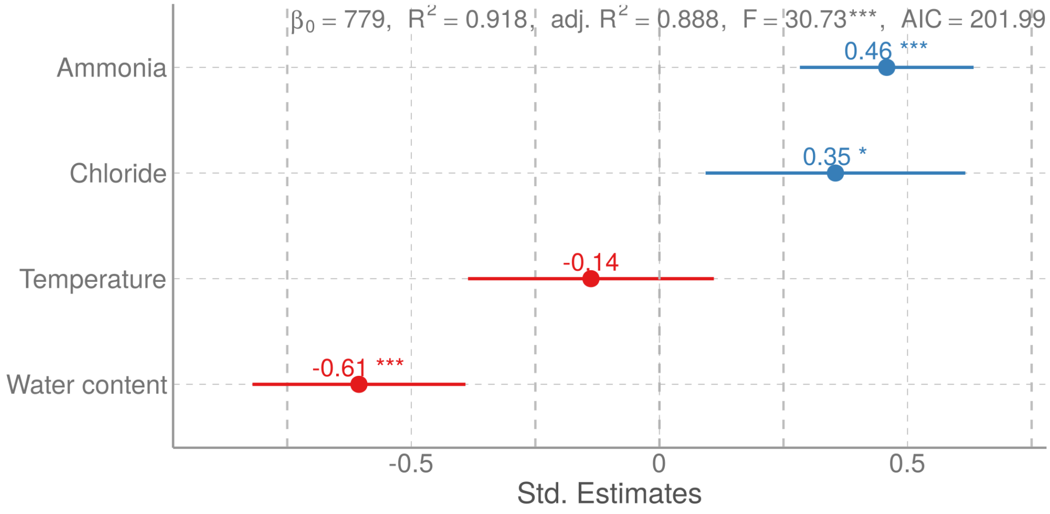

(a)

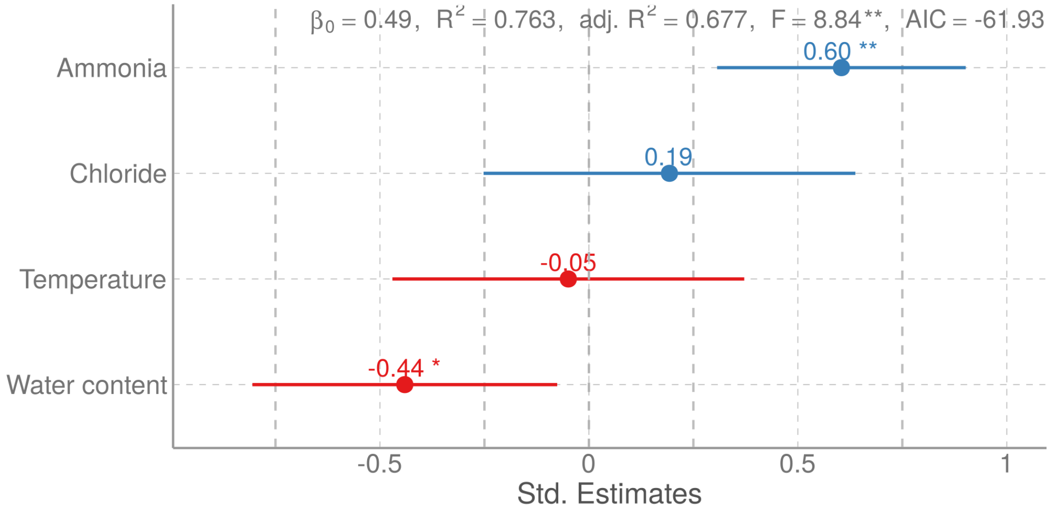

(b)

Figure S6. Graphical representation of the results of the linear model fit of the chemical parameters to richness (a) and evenness (b) as explanatory variable.

Table S1: Used primers

Analysis	Primers	Sequence (5' to 3')
Quantitative PCR	S-D-Bact-0341-b-S-17	CCTACGGGNGGCWGCAG
	S-D-Bact-0515-a-A-19	TTACCGCGGCTGCTGGCAC
T-RFLP	S-D-Bact-0341-b-S-17-FAM	CCTACGGGNGGCWGCAG-FAM
	S-D-Bact-0907-a-S-20	AAACTYAAARRAATTGACGG
Illumina sequencing	S-*-Univ-0515-a-S-19	GTGCCAGCMGCCGCGGTAA
	S-D-Bact-0787-b-A-20	GGACTACHVGGGTWTCTAAT

Table S1. Primes targeting 16S rRNA gene used in this study (Klindworth et al. 2012)

SI text 1. Soil chemical analysis

About 500 g of the field-collected soil samples was chemically analyzed as previously described (Si et al. 2007). Values of pH and EC were measured in a saturated soil-paste extract. Phosphorus ions were extracted from the soil with $0.5\,\mathrm{mol\,l^{-1}}$ $NaHCO_3$ (Olsen and Watanabe 1957) and analyzed colorimetrically in an auto analyzer (ASX-520 series, Quickchem 8500 series 2; Lachat instruments, Loveland, CO). Potassium was extracted by $CaCl_2$ and measured by flame atomic absorption spectrophotometry (Flame photometer M410; Sherwood Scientific, Cambridge, UK). Chloride in saturated soil paste extract was measured with a chloride meter (Chloride analyzer 926; Sherwood Scientific Ltd, Cambridge UK). Soil organic matter content was estimated by weight loss using the ignition method.

In the figure S1, electrical conductivity and chloride ions changed with the wetting front. Their concentration dropped from around $30\,\mathrm{mg\,kg^{-1}}$ to $10\,\mathrm{mg\,kg^{-1}}$ on days 2 to 4 and then steadily rose back to $30\,\mathrm{mg\,kg^{-1}}$ in tandem with the soil's desiccation (a,e). The soil pH was unchanged throughout the sampling period, mainly due to the desert soil's buffering capacity (c). Phosphorus and potassium ion concentrations in desert soil are usually correlated to plant litter Cross and Schlesinger (1999) and remained largely unchanged in the sampled barren soil (f,h). The amount of ammonium and nitrates in our samples followed previously reported patterns Austin et al. (2004), with ammonia elevated during the wet period and lower in dry soil and nitrates accumulating in the dry soils and depleted during hydration (b,d). Lastly, the amount of total organic carbon was assessed throughout the sampling period; it increased on days 3 and 10 after hydration and in the early desiccation period (g).

SI text 2. Statistical analysis of the soil chemical composition

A null model was constructed first to evaluate the effect of field as an error term using linear mixed model (function lmer in R, package lme4 v1.1-9) (Zeileis and Hothorn 2002). Removing it did not influence the explanatory power of the model as measured by anova comparison (R, function ANOVA package stats v3.2.2). After removing temperature factor, the variance inflation factor (VIF) of all the samples dropped below the acceptable threshold of 5. Visual inspection of homoschedasticity, normality of residuals, Q-Q plot of residuals and residuals against predictors was inspected with the R sjPlot package function sjp.lm type "ma" (Lüdecke and Lüdecke 2016). The model was subsequently reduced to contain only water content, ammonia and chloride ions. The reduced model was not significantly different from the full model as tested with R, function anova. Final model colinearity was < 2, residuals were normally distributed as tested with the Shapiro test (Richness: $W = 0.9516$, $p = 0.516$; Evenness : $W = 0.9716$, $p = 0.865$) (Rovston 1982). Homoscedasticity of residuals was tested with the studentized Breuch Pagan test with R function bptest from the package lmtest v0.9-34 (Breusch and Pagan 1979; Zeileis and Hothorn 2002)(Richness: $BP = 5.967$, $df = 3$, $p = 0.11$; Evenness: $BP = 2.873$, $df = 3$, $p = 0.41$). All of the linear model assumptions were met.

SI text 3. MiSeq sequencing analysis

Sequencing files were analysed with the Qiime pipeline Caporaso et al. (2010). Barcodes and sequencing primers were removed in the sequencing facility. Samples were quality filtered to the phred score of 25. Subsequently the chimeric sequences were removed with the uchime algorithm (Edgar et al. 2011) using both a reference database (ChimeraSlayer reference database in the Broad Microbiome Utilities version microbiomeutil-r20110519) and de novo chimera detection. Approximately 30% of sequences were removed in this step. The remaining sequences were clustered with the pick_open_reference_otus.py pipeline, using cluster algorithm against a Silva database v119 (Quast et al. 2013). The sequences were custard on the 90% identity level. The resulting dataset was further analysed with the phyloseq package v1.12.2 in R (McMurdie and Holmes 2013).

SI text 4. The modified rough surface patch model

The rough surface patch model (RSPM) is employed and modified to generate physical domains (Kim and Or 2016). In the RSPM, geometrical information of soil structure was averaged with a probability distribution of angular pore sizes and the effective water film thickness was introduced as an indicator of hydration condition that controls substrate diffusion, microbial dis-

persion rates, and aqueous habitat connectivity. In this study, we extended the probability based description from the rough surface domain to the soil profile domain to include gas phase and its diffusion during wetting. The domain for microorganisms is represented as a vertical section of the soil profile comprised with hexagonal patches (Figure SI.1(A)). Each patch (with a size 500μm in this work) is a spatial element of the domain that can be treated as a subdivided region with given soil properties, such as porosity ϕ, and a fractal dimension D for the pore-size distribution. These properties determine representative hydraulic measures essential for microbial life, such as effective water film-thickness, degree of saturation, effective void space volume, and connectivity of aqueous habitats. Locally pre-assigned properties yield spatial heterogeneity of water distribution in the domain at a given relative humidity.

Aqueous phase and gas phase in the physical domain

Following the RSPM, the same approach is used but extended by assuming the smooth surface region in RSPM as a cube and the surface pore as a square pyramid void (Figure SI.1(A)). It allows to calculate the local porosity ϕ of each patch (a small volume of bulk soil) to describe the soil type and roughness (described with the fractal dimension D and the surface porosity Φ) and its saturation degree at the given matric potential. The relation between the surface porosity in the RSPM and the total porosity of the soil profile in the modified RSPM can be written as following:

$$\phi = 1 - \frac{\int_{r_{\min}}^{r_{\max}} \left(\frac{2}{3}pH(r) + sr\right) r^2 \mathcal{N}(r)dr}{\int_{r_{\min}}^{r_{\max}} \left(pH(r) + sr\right) r^2 \mathcal{N}(r)dr} \tag{SI.1}$$

$$\equiv 1 - \frac{\int_{r_{\min}}^{r_{\max}} \left(\frac{2}{3}p + s\right) r^3 \mathcal{N}(r)dr}{\int_{r_{\min}}^{r_{\max}} \left(p + s\right) r^3 \mathcal{N}(r)dr} = 1 - \left\{\frac{2}{3}\Phi + (1-\Phi)\right\} = \frac{1}{3}\Phi, \tag{SI.2}$$

where $r_{\min}$ and $r_{\max}$ are the cutoff values for the pore size distribution, $H(r)$ is the height of the square pyramid pore with size r (Here, we assumed that the height of each square pyramid pore is the same as its base.), $\mathcal{N}(r) \sim r^{-D}$ is the probability density function of pore size r, p and s are the fraction of pore or solid elements in the space, respectively. We note that this relation could be different when different shape factors for solid and pore are assumed. The saturation degree at the given matric potential ψ_m can be obtained following RSPM.

$$\Theta(\psi_m) = \frac{\int_{r_{\min}}^{r_{\max}} [\Phi\mathcal{V}(r,\psi_m) + (1-\Phi)h_\mu r^2]\mathcal{N}(r)dr}{\int_{r_{\min}}^{r_{\max}} [\Phi\frac{1}{3}r^2H(r) + (1-\Phi)h_\mu r^2]\mathcal{N}(r)dr}, \tag{SI.3}$$

where $\mathcal{V}(r,\psi_m)$ is the amount of water held in the pore with size r at the matric potential ψ_m due to the capillary force and the van der Waals force and h_μ is the absorbed water on the smooth surface (For the detailed explanation, see Kim and Or (2016)).

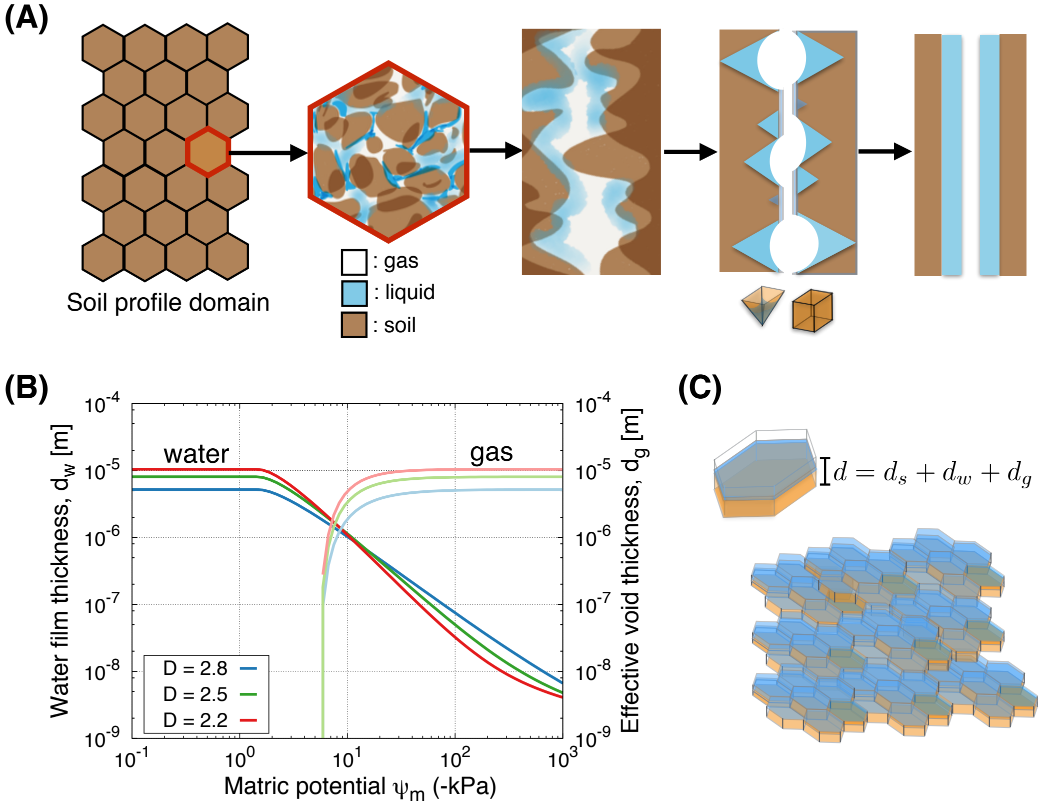

Figure SI.1: A schematic of the physical domain in the modified RSPM. (A) The roughness representation of a surface is extended to the vertical representation of three-dimensional soil profile including the gas phase (Kim and Or 2016). A patch is a collection of roughness elements, cubic solid blocks and square pyramid voids. Using the effective medium assumption, effective thicknesses of each phase are determined. d_s, d_w, and d_g denote the effective thickness of solid, liquid, and gas phases, respectively. The porosity ϕ, the gas content ϵ, and the water content θ of each patch are converted from these effective thickness values. (B) Effective thicknesses for liquid and gas are calculated for three different fractal dimensions for pore size distribution. Strong coloured lines are water film thicknesses and corresponding pastel colours are for the void thicknesses. (C) For each patch, $d_s + d_w + d_g = d$ is given constant (calculated from the pre-assigned porosity) and the hydration condition and the thermal diffusivity are regulated by these effective thicknesses, d_w, d_e, and d_g. As a result, nutrients and heat diffusion between adjacent patches is a function of ψ_m, local relative humidity.

Notations	Parameters	Units	Values
$r_{\min}$	minimum size of pores	m	10^{-7}
$r_{\max}$	maximum size of pores	m	5×10^{-4}
D	fractal dimension for pore size distribution	-	2.8
$\bar{\Phi}$	mean surface porosity	-	0.8
L_p	size of a patch	m	5×10^{-4}

Table SI.1: Parameters used to generate roughness domain for the modified RSPM

The total amounts of solid (soil grains), liquid (water held by capillarity and van der Waals force), and gas phases are mapped to the effective thicknesses to describe the transport properties between patches in the profile. Effective thicknesses of solid, liquid, and gas are d_s, d_w, and d_g, respectively:

$$d_s = (1-\phi)d \tag{SI.4}$$

$$d_w(\psi_m) = \phi\Theta(\psi_m)d \tag{SI.5}$$

$$d_g(\psi_m) = \phi(1-\Theta(\psi_m))d \tag{SI.6}$$

where d is the total thickness of each patch for heat and solute transport and it can be calculated following equation:

$$d - d_s = \phi d = \frac{\int_{r_{\min}}^{r_{\max}} [\Phi \frac{1}{3} r^2 H(r) + (1-\Phi) h_\mu r^2] \mathcal{N}(r) dr}{\int_{r_{\min}}^{r_{\max}} r^2 \mathcal{N}(r) dr}. \tag{SI.7}$$

In this research, the hydration condition (wetting and drying event) is controlled with the matric potential ψ_m mapped from the water contents ($\theta(\psi_m) \equiv \Theta(\psi_m)\phi$). The heterogeneity of the domain was achieved by assigning a set of $\{\Phi, D\}$ for an individual patch. To match the type of soil investigated in the field (Muñoz Castelblanco et al. 2012), we assumed the fractal dimension of the pore-size distribution of loam soil 2.8 (corresponds to a fractional Brownian surface with the Hurst exponent $H = 0.2$) and the mean porosity to be $\bar{\phi} \approx 0.3$ (Tyler and Wheatcraft 1992; Wang et al. 2005; Huang et al. 2006). This means that the probability that a point on the domain belongs to a pore with size X in the interval $[r, r+\delta r]$ is;

$$\Pr[r \leq X \leq r + \delta r] \sim \bar{\Phi} r^{-(D-1)} \delta r. \tag{SI.8}$$

Parameters used to generate the physical domains for the simulations are given in Table S1.

Diffusion processes through the profile

In the physical domain, we averaged microscopic details of the pore distribution and assumed that hydration conditions are represented with effective water film thickness and degree of saturation. The diffusion processes in this work is described as below to calculate local substrate concentration, $C(\vec{r}, t)$,

$$\frac{\partial C(\vec{r,t})}{\partial t} = \nabla \cdot (D(\vec{r}) \nabla C(\vec{r}, t)) - \text{Sink terms} + \text{Source terms} \tag{SI.9}$$

where $D(\vec{r})$ is the apparent diffusion coefficient defined from the effective film thickness distribution of adjacent patches and the effective diffusion coefficient including tortuosity as a function of porosity and water contents. The net flux between two adjacent patches (for example, patch 1 and patch 2) due to diffusion is calculated as following:

$$\vec{J}_{1\to 2} = -\frac{2D_{\text{eff}}(\vec{r_1})D_{\text{eff}}(\vec{r_2})}{D_{\text{eff}}(\vec{r_1}) + D_{\text{eff}}(\vec{r_2})} \min[d_w(\vec{r_1}), d_w(\vec{r_2})] \frac{C(\vec{r_2}) - C(\vec{r_1})}{L_p}. \tag{SI.10}$$

To calculate the flux between heterogeneous medium, we have chosen the harmonic mean of diffusion coefficient and the minimum value of water film thicknesses between neighbouring patches (for the details, see Kim and Or (2016)). The effective diffusion coefficient of a patch is given following the Milington-Quirk tortuosity model (Milington and Quirk 1961).

$$D_{\text{eff}}(\vec{r}) = D_0 \frac{\theta(\vec{r})^2}{\phi(\vec{r})^{4/3}} \tag{SI.11}$$

where D_0 is the diffusion coefficient of the substrate in bulk water and $\theta(\vec{r})$ and $\phi(\vec{r})$ are the water content and the porosity of the patch at $\vec{r}$. In Equation (SI.9), the second and third terms on r.h.s. indicate sink and source of substrates as reaction terms. Source terms are the mass transfer from the gas phase or the input of the source as boundary conditions and sink terms are the consumption by microorganisms.

Furthermore, we included dynamics of oxygen profile driven by diffusion in the gas phase and its input into the liquid phase as the dissolved oxygen. Aerobic bacteria are assumed to uptake the oxygen in the dissolved form (modelled as obligate aerobes) and anaerobic bacteria are inhibited by the local concentration of dissolved oxygen (modelled as obligate anaerobes). However, in the model, combining IBM and the gas diffusivity of oxygen through the soil profile is challenging as the time scales of these processes differ in order of magnitudes. For example, while the growth of a cell is in the order of hours, $\approx 10^3$ seconds, the gas diffusion through the domain (in this work, depth of 5 cm) is in the order of $\approx 10^{-2}$ seconds. Using the time scale of gas diffusion for all processes in the model is not plausible due to the computational time limit. Thus, we did not explicitly solve the gas diffusion for the oxygen source. Instead, we combined

the Henry's law and percolation theory: Firstly, for the mass transfer between gas and liquid, Henry's law was applied;

$$C_O(\vec{r})^* = H_{cc}(T(\vec{r}))C_O^g(\vec{r})^* \tag{SI.12}$$

where $C_O(\vec{r})^*$ is the local concentration of dissolved oxygen in the liquid phase, $C_O^g(\vec{r})^*$ is the concentration of oxygen in the gas phase (converted from the partial pressure of the oxygen in the atmosphere) at equilibrium, and $H_{cc}(T(\vec{r}))$ is the dimensionless Henry's constant when temperature is given as T at the position $\vec{r}$. The temperature dependency of Henry's constant is described as $H_{cc} = H_{cc}^{\Theta} \exp\left(-\frac{\Delta_{\mathrm{soln}} H}{R}\left(\frac{1}{T} - \frac{1}{T^{\Theta}}\right)\right)$ where $\Delta_{\mathrm{soln}}H$ is the enthalpy of solution, R is the gas constant, T is absolute temperature, and Θ refers to standard condition ($T^{\Theta} =$ 298.15K) (Sander 1999). This assumption holds during entire simulations, as the soil matrix is characterised with large specific surface area and thin water film thickness (high mass transfer rate). Secondly, the invasive percolation of gas phase from the top of the domain through the profile is considered during the desiccation period. We assumed the gas percolation threshold based on the gas content of each patch. When the gas content of a patch exceeds a certain value (i.e. $\epsilon(\vec{r}) > \epsilon_c$. In this work we used $\epsilon_c = 0.243$ assuming the threshold of 3 dimensional body-centred cubic lattice (Sykes and Essam 1964)), we assign the patch as a percolating patch.

Environmental conditions: temperature

The heat transport equation can be used to calculate the soil temperature profile $T(\vec{r}, t)$ (in the absence of fluid motion):

$$c_v(\vec{r})\frac{\partial T(\vec{r}, t)}{\partial t} = \nabla \cdot (\lambda(\vec{r})\nabla T(\vec{r}, t)) \tag{SI.13}$$

where $c_v(\vec{r})$ is the local heat capacity and $\lambda(\vec{r})$ is the local thermal conductivity at $\vec{r}$. From the local information of volume fractions and densities of solid, water, and gas, the soil volumetric heat capacity can be written:

$$c_v(\vec{r}) = \rho_s(1 - \phi(\vec{r}))c_s^s + \rho_w\theta(\vec{r})c_s^w + \rho_g\epsilon(\vec{r})c_s^g, \tag{SI.14}$$

where c_s is the specific heat capacity per unit mass, ρ is the density, and s, w, and g for each variables denote soil minerals, water, and gas. The volume fraction of each phase is given as $(1 - \phi)$, θ, ϵ for solid, liquid, and gas, respectively. In this equation, the proportion of organic matter (such as EPS, or microbial cells) are ignored for the thermal properties. Each volume fraction is determined by the hydration condition, therefore $c_v(\vec{r})$ varies following the water contents (Figure S4). The effective thermal conductivity at $\vec{r}$, $\lambda(\vec{r})$ is given as a harmonic mean of three conductivities, λ_s, λ_w, and λ_g from different phases.

$$\lambda(\vec{r}) = \left(\frac{1 - \phi(\vec{r})}{\lambda_s} + \frac{\theta(\vec{r})}{\lambda_w} + \frac{\epsilon(\vec{r})}{\lambda_g}\right)^{-1}. \tag{SI.15}$$

However, the thermal diffusivity in soil is also in the order of $\approx 10^{-6} m^2/s$, hence the coupling the microbial growth is not plausible. For the temperature, we solved the homogenous 1 dimensional domain (over the depth) at the varying hydration condition and applied the solution to the profile as boundary conditions. Considering that the soil crust (with 2mm thickness) was discarded and the sample was collected from top 5cm, we applied soil crust (fine soil structure with $D = 2.9$ and $\Phi = 0.9$) at the top 2mm and used Loess soil texture ($D = 2.8$ and $\Phi = 0.8$ same as the value we used in the main text) for the below crust. Boundary condition at the air-soil interface is assumed to be in the interfacial isothermal condition.

$$T(z = 0, t) = T_{\mathrm{air}}(t) \tag{SI.16}$$

where $T_{\mathrm{air}}(t)$ is assigned from the air temperature records of LTER where our field measurements were conducted. Considering the thermal damping depth of loess soil is around 10 cm, we modelled up to 15 cm with the zero heat flux boundary condition at the bottom of the domain. The calculated solution at the three selected depths are plotted in Figure SI.2. To sum up the

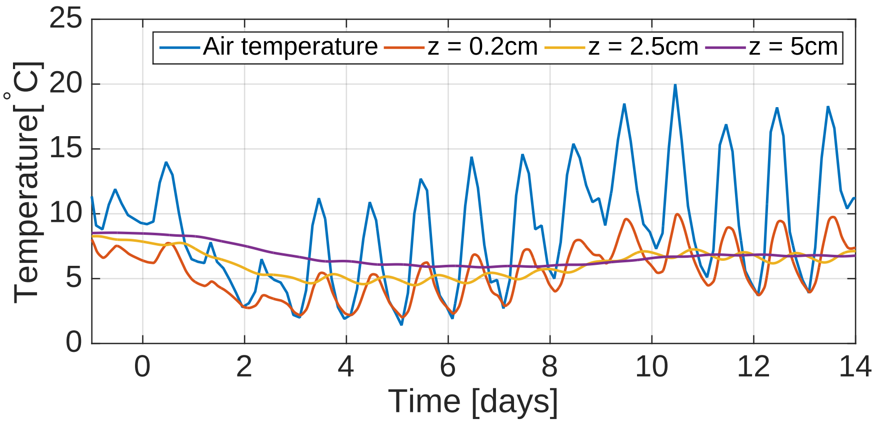

Figure SI.2: Calculated soil temperature profile from the air temperature records of LTER. Prediction of soil temperature profile during the field observation are plotted and the time corresponds to the time of field measurements in the main text. The blue line indicates the air temperature. The predicted soil temperature is given for depths, z = 0.2 (red), 2.5 (yellow), and 5 cm(purple). Only top 5cm of the soil is sampled after discarding the soil crust with 2mm thickness.

mechanistic model for microbial populations in soil, a schematic of the model and its boundary conditions for hydration conditions, substrate and heat diffusion are given in Figure SI.3.

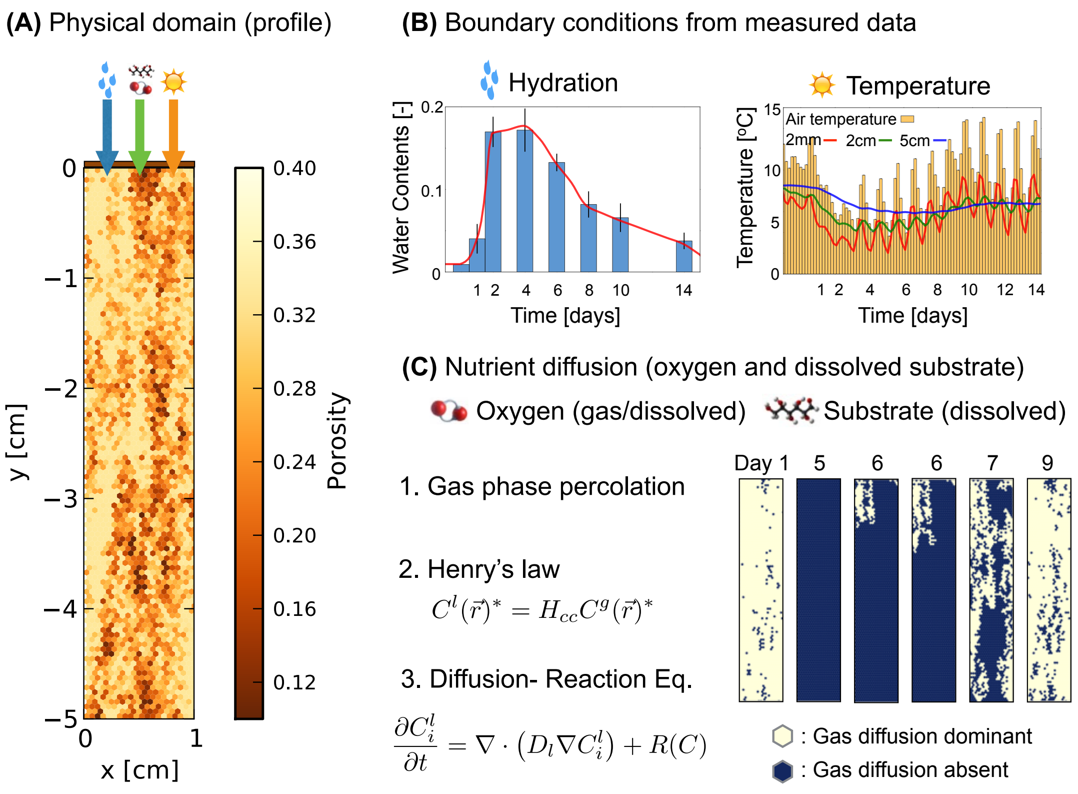

Figure SI.3: A schematic of the physical domain of the mechanistic model used in this work.
(A) Physical domain is a soil profile with pre-assigned distribution of structural properties, such as porosity and fractal dimension (in this figure, fractal dimension $D = 2.8$ is used). The domain mimics the soil sample collected from the field (up to 5 cm depth from the surface).
(B) Hydration and temperature conditions are used as dynamic boundary conditions through the entire simulations. For the hydration condition, measured gravimetric water contents (blue bars) were linearly interpolated (the red line) and mapped to the matric potential of the entire domain. By using the air temperature records of LTER, we calculated the soil temperature (combined with the hydration condition changes) over the profile. Orange bars are the record of air temperature, red, green, blue lines are the soil temperatures at depth 2mm, 2cm, and 5cm, respectively.
(C) Dissolved nutrient distributions are solved with diffusion-reaction equations. Especially, the oxygen source in the profile (for aerobic cells) is the partition of gas and liquid phases and the model solves the input of dissolved oxygen from gas phase by combining gas percolation and Henry's law.

Individual based description of microbial growth on the heterogeneous domain

Microbial activity was added to the modified RSPM by using the IBM (Individual based model) (Kim and Or 2016). The RSPM allows assigning local environmental conditions and the IBM examines cell-level response to it. Since the IBM is suitable for describing bacterial behaviour in heterogenous and time-variant environment, combining these two models can be interpreted as an *in silico* experiment based on physical models. Each individual in the IBM represents microbial cell possessing intrinsic parameters that indicate its physiological characteristics, such as cell-size, specific growth rate, nutrient affinity, etc. The model tracks life history of each individual such as interactions with other cells and growth history at a single-cell point of view (see reviews by Ferrer et al. (2008); Hellweger and Bucci (2009)). As an addition to previous IBM models, our model is spatially explicit and includes motility of cells as they are able to actively explore the domain. The motility of a cell is regulated by the chemotactic response to substrate concentration and the swimming velocity on capillary surfaces. For its locomotion and net displacement, we used a biased random walk approach on the rough surface.

A reaction-diffusion equation was used in the model to obtain the nutrient distribution over time:

$$\frac{\partial C_j(\vec{r},t)}{\partial t} = \nabla \cdot (D_j(\vec{r},t)\nabla C_j(\vec{r},t)) - \frac{1}{V_w(\vec{r},t)}\sum_{i=1}^{N(\vec{r})}\frac{\mu_i(\vec{r})}{{Y_{\max}}_j^i}b_i(t) + S_j(\vec{r}), \qquad \text{(SI.17)}$$

where $C_j(\vec{r},t)$ is the local concentration of substrate j, $D_j(\vec{r},t)$ is the local diffusion coefficient, and $V_w(\vec{r},t)$ is the amount of water in a given patch at time t. The second term on the right-hand side is the reaction term calculating the total substrate consumption in the patch. $N(\vec{r})$ is the total number of individual cells at $\vec{r}$, ${Y_{\max}}^i$ is the maximum growth yield of taxon i on the substrate j, $b_i(t)$ is the biomass, and $\mu_i(\vec{r})$ is the growth rate of cell i. The last term $S_j(\vec{r})$ is the source term of the substrate j.

Each cell consumes several chemical species that are obligatory for its growth, in this study oxygen and dissolved carbon following their physiological differences. We assign a growth rate of a taxon (or microbial species) i with multiple limiting substrates j by using Monod-type growth kinetics as a function of the substrate concentration field (Monod 1942, 1949):

$$\mu_i(\vec{r}) = \mu_{\max,i}\min[f_i^1(\vec{r}), f_i^2(\vec{r}), \cdots] \qquad \text{(SI.18)}$$

where $f_i^j = \frac{C_j}{K_{S,i}^j + C_j}$ (when nutrient j is a substrate for the growth) or $f_i^j = \frac{K_{I,i}^j}{K_{I,i}^j + C_j}$ (when nutrient j is a inhibitor for the growth) and ${\mu_{\max}}^i$, $K_S^i/K_{I,i}^j$ are the maximum growth rate and half-saturation/inhibition constant of cell i, respectively. Specifically, in this study, we introduce two different groups of microorganisms, aerobically growing group and anaerobically growing group. Aerobes are simply assigned as a group that utilises oxygen and carbon source for growth.

On the other hand, growth of anaerobes are inhibited by the presence of oxygen, meaning that only obligate anaerobes are considered. The growth rate of individual cell is calculated as following: For aerobic growth,

$$\mu_i^{ae}(\vec{r}) = \mu_{\max,i}\min[\frac{C_C(\vec{r})}{K_{S,i}^C + C_C(\vec{r})}, \frac{C_O(\vec{r})}{K_{S,i}^O + C_O(\vec{r})}] \tag{SI.19}$$

For anaerobic growth,

$$\mu_i^{an}(\vec{r}) = \mu_{\max,i}\min[\frac{C_C(\vec{r})}{K_{S,i}^C + C_C(\vec{r})}, \frac{K_{I,i}^O}{K_{I,i}^O + C_O(\vec{r})}] \tag{SI.20}$$

where $C_C(\vec{r})$ is the local concentration of the carbon source and $C_O(\vec{r})$ is the local concentration of the dissolved oxygen. We assumed that the individual growth rate at the pore scale is the same as the population growth rate in batch culture (Dai et al. 2013). Accordingly, the growth of an individual cell can be written as:

$$\frac{db_i(t)}{dt} = [\mu_i(\vec{r}) - m_i]\, b_i(t) \tag{SI.21}$$

where m_i is the maintenance rate of cell i.

Characteristics of multiple taxa were assigned before the inoculation on the RSPM. To avoid the complex definition of bacterial taxa, we defined different bacterial "taxa" based only on their nutrient-consumption patterns. Any other functional diversity or complex trophic interactions were not included in the model. The values used in the simulations are listed in Table SI.2.

Temperature dependent microbial growth

In the model, we combined our growth model with a temperature dependent growth model using the Arrhenius equation (Schoolfield et al. 1981) to see the effect of temperature in population dynamics. The level of adaptation to the temperature might vary among different organisms, however, we did not consider it and assumed that all microbial cells follow the same activation energy and the optimal temperature encapsulated in the maximum growth rate to reduce the complexity. The temperature dependency on the maximum growth rate of a cell at temperature T is assigned as following (Schoolfield et al. 1981):

$$\tilde{\mu}_i(T) = \mu_{\max,i}\left[\frac{\frac{T}{T_\Theta}e^{\frac{\Delta H_\Theta}{R}\left(\frac{1}{T_\theta}-\frac{1}{T}\right)}}{1+e^{\frac{\Delta H_L}{R}\left(\frac{1}{T_L}-\frac{1}{T}\right)}+e^{\frac{\Delta H_H}{R}\left(\frac{1}{T_H}-\frac{1}{T}\right)}}\right], \tag{SI.22}$$

where T_Θ is the reference temperature ($25°C = 298K$) and ΔH_Θ (cal.mol^{-1}) is the activation enthalpy of the reaction. There are two inactivation regimes; low temperature inactivation and

Notations	Parameters	Units	Values
${\mu_{\max}}^i$	specific growth rate	hr^{-1}	$0.3 \sim 1.0^{ae,\#}$ / $0.6 \sim 1.8^{an,\#}$
$Y_{\max}$	apparent yield	$fg.fg^{-1}$	0.44^{ae} / 0.088^{an}
m	maintenance rate	-	0.041^{ae} / 0.0041^{an}
$K^C_{S,i}$	half-saturation constant	$mg.l^{-1}$	$0.001 \sim 0.1^{\#}$
$K^O_{S,i}$	half-saturation for O_2	$mg.l^{-1}$	2.3^a
$K^O_{I,i}$	inhibition constant for O_2	$mg.l^{-1}$	0.05^b
$\bar{V_B}$	median cell volume	fl	0.4
$V_{\mathrm{B,d}}$	cell volume at division	fl	$2\bar{V_B}/1.433$
$V_{\mathrm{min,d}}$	minimal active cell volume	fl	$\bar{V_B}/5$
ρ	cell density (dry mass)	$fg.fl^{-1}$	290
R	size of microbial cells	μm	1
D^C_0	substrate diffusion coefficient	$mm^2.hr^{-1}$	2.2
D^O_0	oxygen diffusion coefficient	$mm^2.hr^{-1}$	7.2
v_0	cell velocity at bulk water	$mm.hr^{-1}$	3.6
χ_0	chemotactic sensitivity	$mm^2.hr^{-1}$	12.8
$C(z=0)$	substrate concentration at the boundary	$g.l^{-1}$	1

Table SI.2: Parameters for individual-based modeling (IBM)

^{ae} Values assigned for the aerobically growing cells; ^{an} Values assigned for the anaerobically growing cells: Assuming that anaerobic processes are costly; oligotroph-like aerobes and copiotroph-like anaerobes based on observed values (Pirt 1965; Heijnen 1999; Stouthamer 2012). We note that higher maximum growth rates for anaerobes were chosen to compensate the strong inhibition of oxygen

$^{\#}$ $\mu_{\max}$ and $K^C_{S,i}$ are different for each taxon. $\mu_{\max}$ are chosen uniformly spaced values and $K^C_{S,i}$ are logarithmically spaced values in the given ranges

^{a} The growth of obligate aerobes ceases at 25% of atmospheric level

^{b} The growth of obligate anaerobes is inhibited when oxygen concentration is higher than 0.5% of atmospheric level

Notations	Parameters	Units	Values
ΔH_{Θ}	enthalpy of activation	kJ	-5.43
ΔH_L	enthalpy change for inactivation at low T	kJ	-141.1
ΔH_H	enthalpy change for inactivation at high T	kJ	687.9
T_L	low T inactivation	K	283*
T_H	high T inactivation	K	314.7

Table SI.3: Parameters used for the temperature dependent growth (Schoolfield et al. 1981; Zwietering et al. 1991), * the enzyme inactivation criterion for the low temperature was modified in this model assuming that microorganisms are adapted to the mean soil temperature

high temperature inactivation that are denoted with subscripts, L and H. From the spatially resolved temperature profile from Equation (SI.13), we modified the microbial growth rate accordingly. The used parameters for the temperature dependent growth is given in Table SI.3.

Active cells and potentially active cells before and after wetting event

The model aimed at describing microbial community behaviour under a wide range of matric potential, changes from several kilopascals to megapascals; in other words, the domain can be exposed to very wet conditions after a rainfall event and to very dry conditions after prolonged desiccation. As the soil dries after the rainfall event, the effective water-film thickness can be reduced from 10^{-5} m to 10^{-9} m. This implies that the amount of nutrient flux to a certain location will also be reduced by several orders of magnitude due to thinning of the water film. In the model, this suppresses microbial growth and leads to taxa extinct in the long term. To prevent complete extinction of taxa during simulations with the model, when a cell is starved (i.e., negative growth rate), we count these cells as potentially active cells (Blagodatskaya and Kuzyakov 2013). This enabled us to track changes in microbial interactions in different hydration states without allowing irreversible extinction of certain taxa and to retain rare members in the microbial community while focusing on the main populations retain rare members in the microbial community while focusing on the main populations during the hydration cycles. Therefore, essentially the model describes dynamics of a community with the constant richness while it tracks the relative abundance changes of the populations.

References

Roey Angel. Total Nucleic Acid Extraction from Soil. *Protoc. Exch.*, October 2012. doi: 10.1038/protex.2012.046. URL `http://dx.doi.org/10.1038/protex.2012.046`.

Amy T Austin, Laura Yahdjian, John M Stark, Jayne Belnap, Amilcare Porporato, Urszula Norton, Damián a Ravetta, and Sean M Schaeffer. Water pulses and biogeochemical cycles in arid and semiarid ecosystems. *Oecologia*, 141(2):221–35, October 2004. ISSN 0029-8549. doi: 10.1007/s00442-004-1519-1. URL `http://www.ncbi.nlm.nih.gov/pubmed/14986096`.

Evgenia Blagodatskaya and Yakov Kuzyakov. Active microorganisms in soil: critical review of estimation criteria and approaches. *Soil Biology and Biochemistry*, 67:192–211, 2013.

Trevor S Breusch and Adrian R Pagan. A simple test for heteroscedasticity and random coefficient variation. *Econometrica: Journal of the Econometric Society*, pages 1287–1294, 1979.

J Gregory Caporaso, Justin Kuczynski, Jesse Stombaugh, Kyle Bittinger, Frederic D Bushman, Elizabeth K Costello, Noah Fierer, Antonio Gonzalez Peña, Julia K Goodrich, Jeffrey I Gordon, Gavin A Huttley, Scott T Kelley, Dan Knights, Jeremy E Koenig, Ruth E Ley, Catherine A Lozupone, Daniel Mcdonald, Brian D Muegge, Meg Pirrung, Jens Reeder, Joel R Sevinsky, Peter J Turnbaugh, William A Walters, Jeremy Widmann, Tanya Yatsunenko, Jesse Zaneveld, and Rob Knight. QIIME allows analysis of high-throughput community sequencing data. *Nat. Methods*, 7(5):335–336, 2010. ISSN 1548-7091. doi: 10.1038/nmeth.f.303. URL `http://dx.doi.org/10.1038/nmeth0510-335`.

Anne Fernald Cross and William H Schlesinger. Plant regulation of soil nutrient distribution in the northern Chihuahuan Desert. *Plant Ecol.*, 145(Balling 1988):11–25, 1999. doi: 10.1023/A:1009865020145.

Jing Dai, Sung Ho Yoon, Hye Young Sim, Yoon Sun Yang, Tae Kwang Oh, Jihyun F Kim, and Jong Wook Hong. Charting microbial phenotypes in multiplex nanoliter batch bioreactors. *Analytical chemistry*, 85(12):5892–5899, 2013.

Robert C Edgar, Brian J Haas, Jose C Clemente, Christopher Quince, and Rob Knight. Uchime improves sensitivity and speed of chimera detection. *Bioinformatics*, 27(16):2194–2200, 2011.

Jordi Ferrer, Clara Prats, and Daniel López. Individual-based modelling: an essential tool for microbiology. *Journal of Biological Physics*, 34(1-2):19–37, 2008.

Joseph J Heijnen. Bioenergetics of microbial growth. *Encyclopedia of Bioprocess Technology*, 1999.

Ferdi L Hellweger and Vanni Bucci. A bunch of tiny individuals : Individual-based modeling for microbes. *Ecological Modelling*, 220(1):8–22, 2009.

Guan-Hua Huang, Ren-Duo Zhang, and Quan-Zhong Huang. Modeling soil water retention curve with a fractal method. *Pedosphere*, 16(2):137–146, 2006.

M Kim and D Or. Individual-based model of microbial life on hydrated rough soil surfaces. *PloS one*, 11(1):e0147394, 2016.

Anna Klindworth, Elmar Pruesse, Timmy Schweer, Jörg Peplies, Christian Quast, Matthias Horn, and Frank Oliver Glöckner. Evaluation of general 16s ribosomal rna gene pcr primers for classical and next-generation sequencing-based diversity studies. *Nucleic acids research*, page gks808, 2012.

Daniel Lüdecke and Maintainer Daniel Lüdecke. Package 'sjplot'. 2016. URL `http://cran.r-project.org/package=sjmisc`.

Paul J McMurdie and Susan Holmes. phyloseq: an R package for reproducible interactive analysis and graphics of microbiome census data. *PLoS One*, 8(4):e61217, January 2013. ISSN 1932-6203. doi: 10.1371/journal.pone.0061217. URL `http://www.pubmedcentral.nih.gov/articlerender.fcgi?artid=3632530&tool=pmcentrez&rendertype=abstract`.

RJ Milington and JP Quirk. Permeability of porous media. *Nature*, 183:387–388, 1961.

Jacques Monod. Recherches sur la croissance des cultures bacteriennes. *Hermann and Cie, Paris.*, 1942.

Jacques Monod. The growth of bacterial cultures. *Annual Reviews in Microbiology*, 3(1):371–394, 1949.

JA Muñoz Castelblanco, JM Pereira, P Delange, and YJ Cui. The water retention properties of a natural unsaturated loess from Northern France. *Geotechnique*, 62(2):95–106, 2012. URL `http://www.icevirtuallibrary.com/content/article/10.1680/geot.9.P.084`.

Sterling R. Olsen and Frank S. Watanabe. A Method to Determine a Phosphorus Adsorption Maximum of Soils as Measured by the Langmuir Isotherm1. *Soil Sci. Soc. Am. J.*, 21(2):144, June 1957. ISSN 0361-5995. doi: 10.2136/sssaj1957.03615995002100020004x. URL `https://dl.sciencesocieties.org/publications/sssaj/abstracts/21/2/SS0210020144`.

SJ Pirt. The maintenance energy of bacteria in growing cultures. *Proceedings of the Royal Society of London B: Biological Sciences*, 163(991):224–231, 1965.

Christian Quast, Elmar Pruesse, Pelin Yilmaz, Jan Gerken, Timmy Schweer, Pablo Yarza, Jörg Peplies, and Frank Oliver Glöckner. The SILVA ribosomal RNA gene database project: improved data processing and web-based tools. *Nucleic Acids Res.*, 41:D590–6, 2013. ISSN 1362-4962. doi: 10.1093/nar/gks1219. URL `http://www.pubmedcentral.nih.gov/articlerender.fcgi?artid=3531112&tool=pmcentrez&rendertype=abstract`.

P Rovston. Algorithm as 181: The w test for normality. *Applied Statistics*, 31:176–180, 1982.

Rolf Sander. Compilation of henry's law constants for inorganic and organic species of potential importance in environmental chemistry, 1999.

RM Schoolfield, PJH Sharpe, and CE Magnuson. Non-linear regression of biological temperature-dependent rate models based on absolute reaction-rate theory. *Journal of theoretical biology*, 88(4):719–731, 1981.

BC Si, RG Kachanoski, WD Reynolds, MR Carter, and EG Gregorich. Analysis of soil variability, 2007.

Adriaan Hendrik Stouthamer. *Quantitative aspects of growth and metabolism of microorganisms*. Springer Science & Business Media, 2012.

MF Sykes and JW Essam. Critical percolation probabilities by series methods. *Physical Review*, 133(1A):A310, 1964.

Scott W Tyler and Stephen W Wheatcraft. Fractal scaling of soil particle-size distributions: analysis and limitations. *Soil Science Society of America Journal*, 56(2):362–369, 1992.

Kang Wang, Renduo Zhang, and Fuqin Wang. Testing the pore-solid fractal model for the soil water retention function. *Soil Science Society of America Journal*, 69(3):776–782, 2005.

Achim Zeileis and Torsten Hothorn. Diagnostic checking in regression relationships. 2002.

MH Zwietering, JT De Koos, BE Hasenack, JC De Witt, and K Van't Riet. Modeling of bacterial growth as a function of temperature. *Applied and Environmental Microbiology*, 57(4):1094–1101, 1991.
